# The Outlook of Green Building Development in China during the “Fourteenth Five-Year Plan” Period

**DOI:** 10.3390/ijerph20065122

**Published:** 2023-03-14

**Authors:** Suyang Xue, Jiaming Na, Libin Wang, Shuangjun Wang, Xiaoxiao Xu

**Affiliations:** 1 College of Civil Engineering, Nanjing Forestry University, Nanjing 210037, China; 2 Housing and Real Estate Promotion Center, Jiangsu Provincial Department of Housing and Urban-Rural Development, Nanjing 210008, China

**Keywords:** green building development, qualitative research, 14th Five-Year Plan, China

## Abstract

To achieve the “Double Carbon” target, China is paying increasing attention to green building development. Thus, this study selected 26 regional green building development planning documents that have been put into practice since the implementation of the 14th Five-Year Plan and analyzed different development goals and common development barriers and paths presented in regional documents by conducting qualitative research. After the analysis of common goals and goals with regional characteristics, this study verified that spatial imbalances did exist in the development targets of green building in each region during the “14th Five-Year Plan”, and the development priorities also varied from region to region. Due to the relation between development goals and the current situation, this study can also illustrate the spatial imbalances of the development situation between different regions. The results of this study can assist regional governments in gaining a clear self-positioning to judge whether they keep pace with the national development level of green buildings and encourage them to take measures to guarantee the stable development of green buildings.

## 1. Introduction

As the global warming trend intensifies, the international community has been trying, with the utmost effort, to establish a low-carbon and environmentally friendly model [1]. China is one of the largest carbon dioxide emitters in the world, accounting for nearly 27% of total global emissions [2]. For this reason, President Xi Jinping solemnly proclaimed that China will peak carbon emissions by 2030, aiming for carbon neutrality by 2060 at the General Debate of the 75th Session of the United Nations General Assembly on 22 September 2020 [3]. The introduction of carbon peaking and carbon neutrality goals has set a clear goal and specific timetable for the revolution of energy with the purpose of the energy transition.

According to previous research, 40% of global energy consumption comes from buildings [4]. The construction sector is identified as one of the three main energy-using sectors. The implementation of the “Double Carbon” target is not only a task that must be accomplished by regional governments but also a major issue of close relevance to those working in the construction industry. Then, people come to realize that green building (GB) can be one of the solutions to reduce building energy consumption and reduce carbon emissions from buildings [5]. Furthermore, GB is one of the goals and strategies to achieve sustainable development, and the core elements of the GB design contain sustainable site or location design, energy and environment, and water conservation [6]. Various GB certification systems have been established globally, such as BREEAM, LEED, and CASBEE [7], to evaluate the sustainability of buildings. As one of the paths to realizing the concept of GB, passive buildings, which are also one of the nearly zero-emission building technology systems, can be defined as buildings that guarantee a comfortable indoor climate and reduce the active energy supply through passive technologies [8]. Although the initiation of GB originated in the 1960s and GB in China has good development space, it still has many problems, such as a short development time and lack of development experience [9].

To address this issue, the Ministry of Construction and the Ministry of Science and Technology undertook several Five-Year Plans from the “10th Five-Year” to the “13th Five-Year” plan, all of which considered the development of building energy efficiency and GB [10]. In particular, the latest release of the “Outline of the 14th Five-Year Plan for National Economic and Social Development of the People’s Republic of China and Vision in 2035” has clarified the goal of deeper development for GB and building energy efficiency in China, providing a clear development orientation for the entire country and construction industry [11].

GB is recognized as one of the solutions to use less energy and resources and produce less construction waste [12]. Meanwhile, GB is beneficial to both the environment and health. On the one hand, GB can reduce energy consumption and achieve energy efficiency, catering to the concept of environmental-friendly design [13]. On the other hand, GB can improve the inhabitants’ livability and enhance occupants’ satisfaction, having certain positive effects on health [14]. To ensure the steady development of GB, many countries, such as the United States, the UK, and Japan, have come into a relatively mature stage for the implementation of GB with the application of laws and regulatory systems related to GB [15]. Some scholars also prove that mandatory regulations and incentive-based policies play vital roles in GB implementation [16].

Referring to the experience of foreign governments, the Chinese government highlighted the advantages of the combination of compulsory and motivational approaches when implementing GB [15] and continuously promulgated several policies to promote GB development [10,17]. In particular, the “Five-Year” plan indicates that China will update the development plan for GB every five years, so the concept of sustainable GB development can be put into practice.

Due to the vast territory of China covering different climate zones, there is a geographical imbalance existing in the development of GB [18]. Accordingly, local governments have established many detailed green policies based on the combination of the country’s total goals and the development status in their jurisdictions [19]. Some scholars have found that not only geographical aspects but also economic, technical, and educational aspects are key factors influencing GB development in China [20].

However, few studies tried to systematically investigate GB in China from the perspective of both policy and spatial distribution. To bridge the research gap, this study collects development planning documents for GB in the “14th Five-Year Plan” period set by the Ministry of Housing and Urban–Rural Development of the People’s Republic of China (MOHURD) and the regional Department of Housing and Urban–Rural Development and attempts to answer the differences between different regions in GB development, aiming to shed light on the spatial characteristics of GB development in different regions.

GB can effectively reduce carbon emissions in the construction industry and help the industry achieve the “Double Carbon” target as soon as possible. Therefore, this study aims to reveal how GB in China will develop in the next five years, called the “14th Five-Year” plan, from the perspective of the central government, provinces, autonomous regions, and municipalities. This study collected regional development planning documents about GB, focusing on the analysis of the goals extracted from the documents and clarifying the differences between regional development goals. The novelty of this study is that it provides a “panorama” of the differences between different regions from different perspectives about the development of GB in an effective way, especially from the spatial perspective, and provides regional governments with a relatively clear self-positioning to judge whether the current level of development in each region confirms the level of national development. Based on this aim, three research objectives are formulated as follows:

1. To identify regional targets for GB development during the period of the “14th Five-Year Plan”;
2. To summarize the common barriers and pathways of GB development among different regions;
3. To analyze the spatial characteristics of GB development in different regions in the next five years.

## 2. Research Methods and Process

### 2.1. Qualitative Research

Qualitative research is the main method used in this study. Qualitative analysis focuses on language and meaning rather than quantitative analysis of data, and it emphasizes the detailed examination of specific cases that exist in social life; sometimes, some new hypotheses can be generated [21]. When researchers do not have a comprehensive understanding of what is investigated, they will usually conduct qualitative research at the beginning [22].

**Figure 1:**
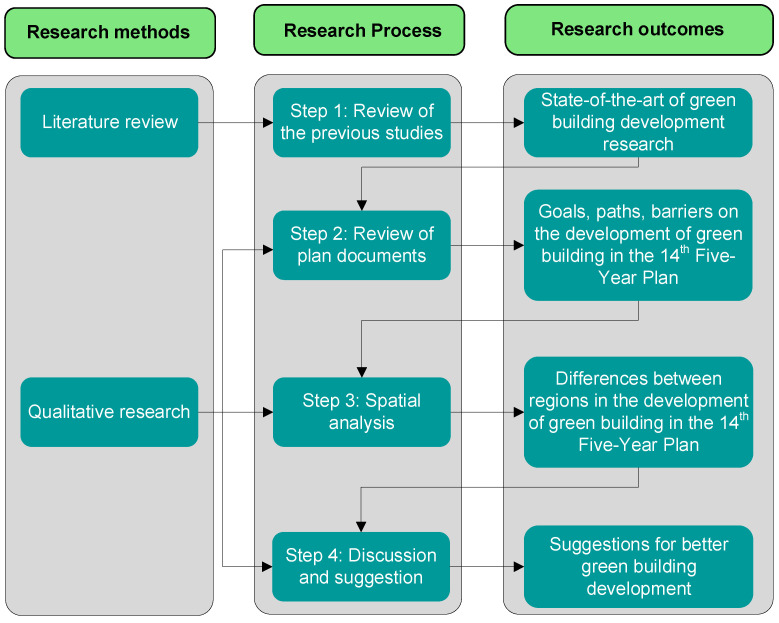
Research methods, process, and outcomes.

Three steps are carried out in this study, namely (1) review of previous studies; (2) review of plan documents from different sectors at different levels; and (3) spatial analysis. The research methods, process, and outcomes are shown in Figure 1.

### 2.2. Research Process

#### 2.2.1. Review of the Previous Studies

The initial literature search is conducted by using the Web of Science, which includes an extensive range of academic publications on GB. A comprehensive search is performed under the topics of “green building” or “green buildings” or “GB”, “green building development” or “green building development in China”, “green building policies” or “green building policies in China”, “China Five-Year plan” or “Five-Year plan for green building”, etc. To avoid the omission of any relevant paper, we expanded the search range to “All Database” and all years till the present. After completing the above process, several papers of close relevance to the identified research topic have been chosen. It is evident from the search results that the current research on green building and green building development is extensive, with a broad research scope. Meanwhile, green building development in China has attracted many scholars in recent years. This study undertakes a systematic review of the literature and collects useful information. Methods and directions are proposed based on these studies.

#### 2.2.2. Review of the Regional Plan Documents

This study performs a comprehensive review of the regional plan documents by searching for the keyword “GB” and “the 14th Five-Year Plan” on official government websites.

In March 2021, the Central People’s Government of the People’s Republic of China published “the 14th Five-Year Plan for National Economic and Social Development of the People’s Republic of China” where the word “green” and “building” were mentioned many times. Then in March 2022, MOHURD issued “the 14th Five-Year Plan for the Development of Energy Efficiency and GB” [23]. The Plan is a more detailed description of the development directions for GB in China from 2021 to 2025 and also sets specific goals for GB in the “14th Five-Year Plan” period. In conjunction with this general outline, the regional Departments of Housing and Urban–Rural Development have proposed new plans and goals for GB development during the “14th Five-Year Plan” period in detail, based on the summary of achievements and difficulties in GB development during the “13th Five-Year Plan” period.

This study covers almost all provinces, municipalities, and autonomous regions of China as far as possible to study their GB development planning documents. At the end of August 2022, 26 regions issued planning outlines for GB development during the “14th Five-Year Plan” period, which are the subjects of this study. Among these 26 regions, 17 regions have issued the official 14th Five-Year Plan for GB development [24,25,26,27,28,29,30,31,32,33,34,35,36,37,38], while the other 9 regions do not have a document specifically describing GB development targets. The other 9 regions have compiled a separate section in planning documents for urban-rural housing development or construction industry development to briefly describe GB development goals [39,40,41,42,43,44,45,46,47]. Liaoning, Henan, Tibet, Xinjiang, Ningxia, Hongkong, Macao, and Taiwan are excluded from this study due to the lack of documents on GB development goals. The reason for selecting a sufficient number of regions for this study is to cover as many regions as possible throughout China, aiming to better reflect the development goals of GB in China during the “14th Five-Year Plan” period. Other regions with a lack of targets for GB development can take these 26 regions’ clear and official planning documents as a reference and prepare their own GB development documents.

#### 2.2.3. Spatial Analysis

In addition to the relevant literature and planning documents, it is necessary to address the differences between different regions in the development of GB during the “14th Five-Year Plan” period. Due to the vast territory and the complex geographical and climatic conditions of China, each region will certainly refer to regional characteristics when preparing its planning outline to set the most appropriate development targets for the region, leading to the targets with regional characteristics, so some scholars, such as Zou et al. [18] and Wang and Jia [48], have found that spatial distribution differences do exist in GB development in China. Therefore, due to the different geographical locations, the selected regions vary from each other in terms of goals for GB development during the “14th Five-Year Plan” period.

## 3. Results

### 3.1. GB Development Goals

In this section, this research presents the GB development goals collected from regional GB planning documents for the 14th Five-Year Plan published on the websites of regional Departments of Housing and Urban–Rural Development (Table 1). Due to a large number of key indicators and the complexity of their presentation, the targets for the 17 regions with specific GB development documents for the “14th Five-Year Plan” are divided into the proportional indicators, area indicators and other indicators in the figures. The limited number of development targets of the other nine regions without a separate GB development document will be shown in a separate figure, respectively. In addition, some quantifiable indicators are chosen from the planning documents to make it easier to compare.

**Figure 2:**
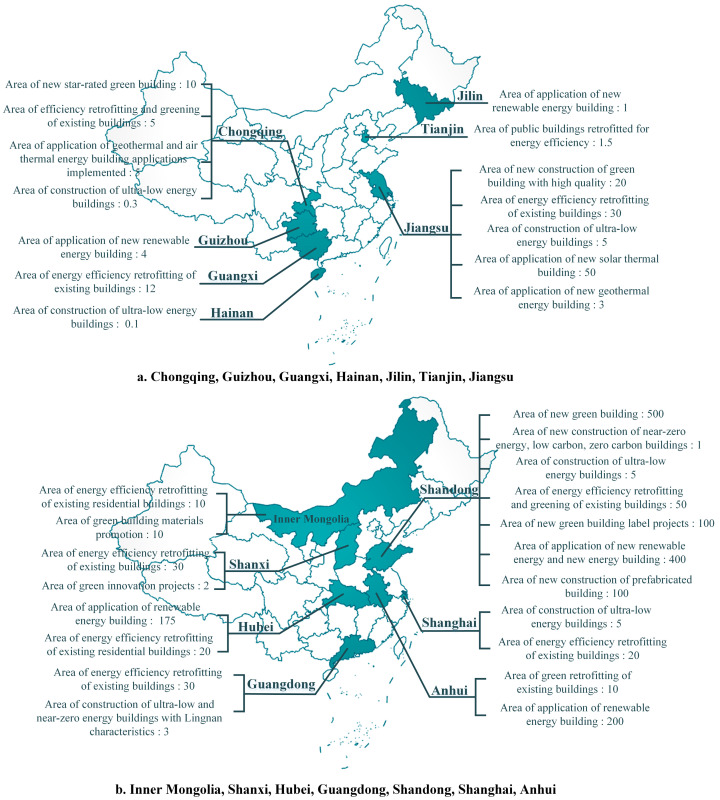
Distribution of area indicators (million square meters).

**Figure 3:**
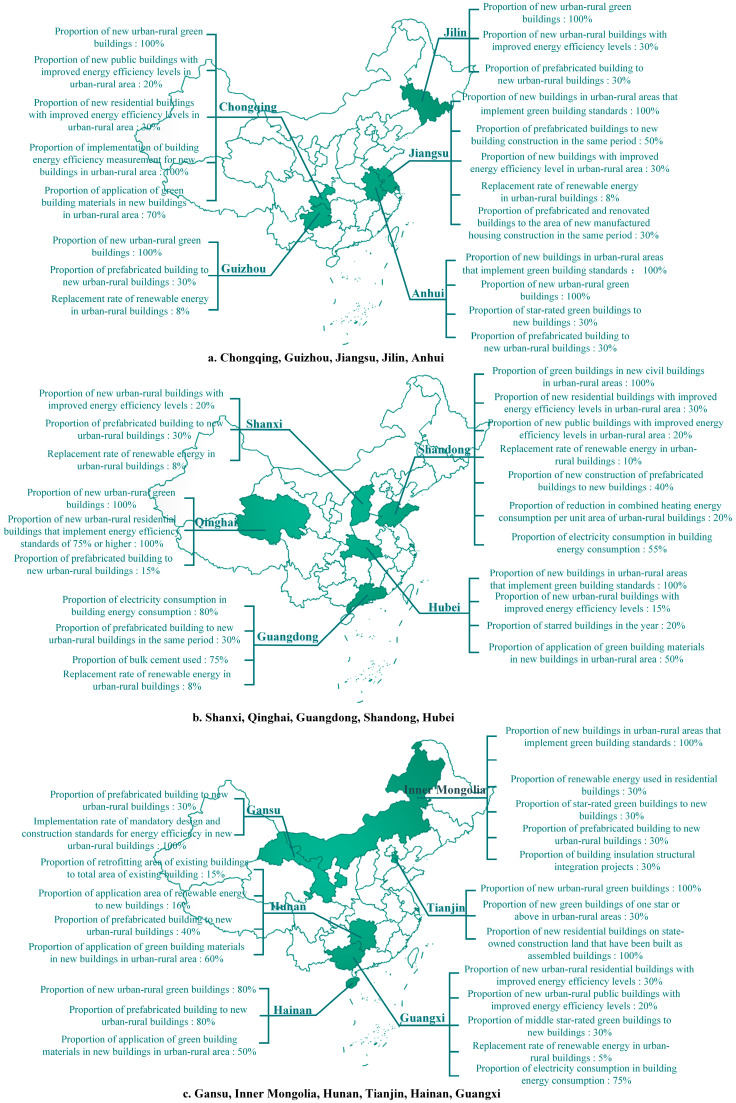
Distribution of proportional indicators.

**Figure 4:**
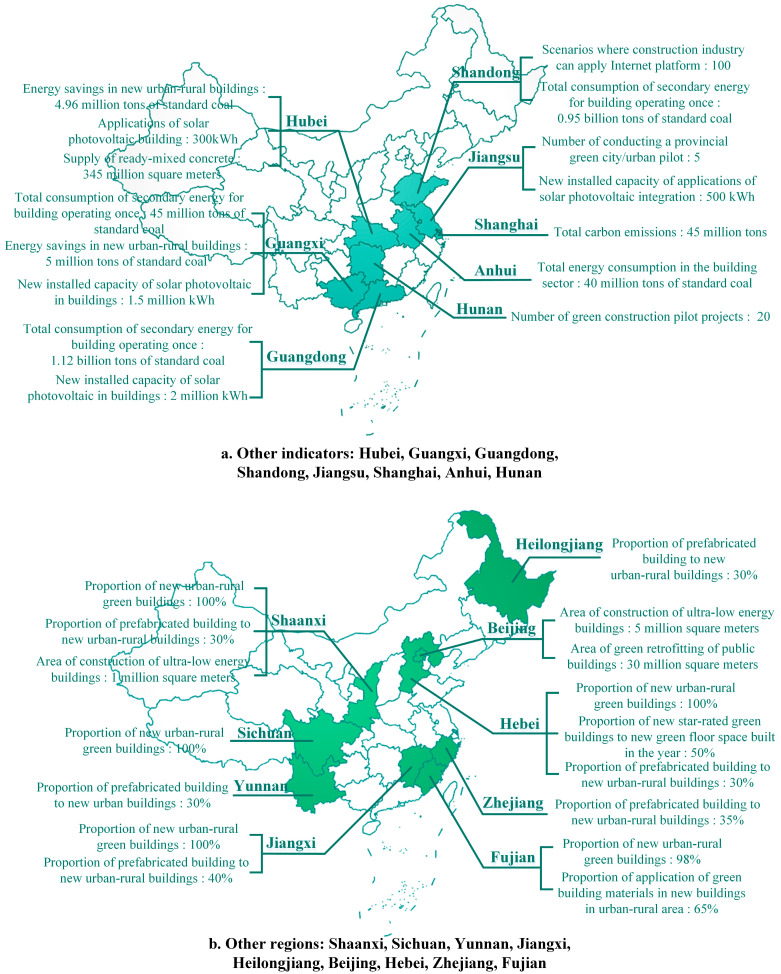
Other indicators and other regions.

The national planning document is very concise and represents national priorities on GB for the next five years, and these brief targets are only guidance for each region. In the USA, green policies at the local level are more detailed than those at the federal and state levels [49]. When the country issues a general planning outline, regional governments will consider the current regional development situation and make a more specific plan in response to the general targets set by the country, which is the concretization of the overall national document. Regional targets for the GB development during the “14th Five-Year Plan” period will be shown in Figure 2, Figure 3 and Figure 4.

### 3.2. Barriers to GB Development

Barriers have always existed and will continue to exist in the development of GB in China. Therefore, after analyzing the policy documents of various regions, this study summarized the shortcomings of GB development at the end of the 13th Five-Year Plan and then concluded five barriers to GB development. Although the 14th Five-Year Plan has been underway for more than one year, the outline of GB development for the 14th Five-Year Plan was released in March 2022 in most regions, and thus deficiencies of the 13th Five-Year Plan development aptly reflect the current state of GB development in China.

#### 3.2.1. Low-Quality GB Development

The quality of GB development needs to be further improved. The quality of GB design is not high and innovative enough, and the quality of construction needs to be improved. The whole process of close supervision needs to be strengthened, and the number of high-star GBs and ultra-low energy consumption buildings is small. Moreover, the speed of energy efficiency retrofitting of existing residential buildings is slowing down due to the lack of supporting policies. Building energy efficiency and GB in rural areas are still in the pilot stage.

The traditional development mode of GB emphasizes quantity and design and neglects quality and operation, making it difficult to obtain high-quality GB. Among the GB built in most regions, only the design stage of these buildings has implemented the concept of green, while the green facilities and equipment are not operated well. The lack of scientific management of operations makes it unavailable to achieve the concept of energy saving and green. Therefore, it is urgent and meaningful to improve the operation management of GB and government supervision.

#### 3.2.2. Low Level of Prefabricated Buildings Development

The development of prefabricated buildings needs to be further promoted. The prefabricated building development in some regions started relatively late. Regional governments and departments are not sufficiently aware of prefabricated buildings, causing slow progress of prefabricated building projects and low acceptance of prefabricated buildings by society and the market. Due to the small scale of prefabricated buildings, the advantages of the reduction of overall cost have not emerged, and construction units are less motivated to develop projects of prefabricated buildings considering incremental costs and short-term interests. The multi-sectoral coordination mechanism for the development of prefabricated buildings has not been completely built. Problems such as the lack of technical standards and management systems and adequate supervision exist in the phases of component production, construction organization, quality acceptance, etc. Briefly, the leading role of regional governments in promoting the development of prefabricated buildings does not function effectively.

#### 3.2.3. Insufficient Awareness and Lack of Sense of Acquisition for GB

The public awareness of GB is not sufficient, and there is a lack of sense of acquisition for GB. During the period of the “13th Five Year Plan”, most regions have further expanded the influence of the green concept through the media and the establishment of demonstration bases for the publicity of GB. However, it is necessary to strengthen the awareness of market entities for GB development and promote the awareness of enterprises and the public for the importance of green development.

In addition, although a certain scale of GB and green ecological residential community projects have been implemented in various regions, problems, such as the unclear comprehensive benefits of GBs; the disconnection between the design, construction, and operation phases; and the poor performance of green technologies, make building users lack the sense of acquisition to GBs.

#### 3.2.4. Imperfect Market Mechanism for GB

Promoting green retrofitting existing buildings and applying renewable energy and ultra-low energy consumption buildings mainly depend on administrative constraints and financial investment. Although the financial support makes a difference, it is still not enough, and the establishment of a long-term market mechanism for the promotion and application of GB is not available. Insufficient enthusiasm from the responsible subjects probably causes problems such as the lack of GBs with high star certification, the low ratio of application of GB materials, and the slow pace of energy efficiency in retrofitting existing residential buildings. The integration of green finance and GB is still in the exploration stage. In addition, the scope of application of market-based measures such as green finance and carbon trading is relatively small, and regulations such as quotas for building energy consumption must be solved. Generally, the working mechanism of government guidance, market promotion, and the participation of all parties needs to be further improved.

#### 3.2.5. Weak Foundation for GB Material Industry Development

The foundation for the GB materials industry development needs to be further improved. Due to the insufficient awareness of GB materials development, the application of GB materials is still based on the promotion of the regime in most regions, and there is a lack of effective government guidance and incentive policies. Simultaneously, the certification of GB materials in most regions is still in the initial stage. GB materials have few categories and a low application ratio, and it is necessary to further improve the types and quantities of certified GB materials. Most importantly, the cultivation and layout of the national GB materials industry need to be enhanced deeply.

### 3.3. Development Paths of GB

**Figure 5:**
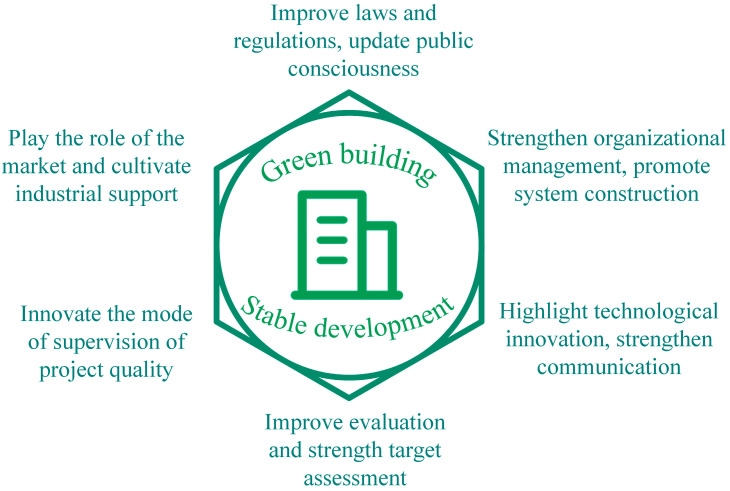
Common development paths of regions.

GB has become a national strategic objective, and every sector of society should make joint efforts to promote the development of GB. The laws and regulations and mass awareness are the foundation, organization, and management are the guarantee, and scientific and technological innovation is the crucial measure (Figure 5).

#### 3.3.1. Improve Laws and Regulations, Update Public Consciousness

Laws and regulations are fundamental in improving GB development [50]. With the goals of carbon peaking and carbon neutrality, the country should promote a system of laws and regulations on building energy efficiency and GB. The country should further intensify law enforcement and ensure that laws and regulations on GB can be put into effect. Central government should guide local governments to formulate or revise relevant local laws and regulations in line with local realities and encourage local governments to develop higher-level local standards for building energy efficiency and GB. Regional Departments of Housing and Urban–Rural Construction should strengthen communication between different departments such as the Development and Reform Commission, Ministry of Finance of the People’s Republic of China, and State Taxation Administration. To promote the synergistic development of green finance and GB, governments should encourage the use of Public–Private Partnerships (PPP) in public services.

The way to update the public’s consciousness is to conduct propaganda and education. Governments at all levels should actively carry out social publicity and education, make full use of various media to publicize the knowledge of GB to the public, improve the understanding of GB, advocate green and low-carbon lifestyles, and consolidate the mass foundation for the high-quality development of GB.

#### 3.3.2. Strengthen Organizational Management, Promote System Construction

The country should optimize the national GB identification and management system, speed up the certification of GB materials, and carry out the pilot project of building energy efficiency evaluation and identification. Under the guidance of the central government, related industries should strengthen data-sharing with each other. Local urban–rural housing construction systems should strengthen the leadership of building energy efficiency and GB, coordinate the role of institutions, implement work responsibilities, and strengthen synergistic cooperation between departments. Regional governments should urge the construction sector to strictly implement the relevant mandatory standards and management regulations for GBs, strengthen the supervision and management of the entire process of new construction projects, and actively promote the information management of the entire construction process.

#### 3.3.3. Highlight Technological Innovation, Strengthen Communication

Regional governments should stimulate the enthusiasm of all sectors of society and encourage and support the introduction of outstanding scientific and technological talents from home and abroad while focusing on training construction professionals and comprehensively improving the quality and competence of the construction science and technology team. At the same time, the country should promote all-round, multi-level, and wide-field cooperation and communication and learn advanced experience from developed countries. It is essential to establish bilateral or multilateral communication and cooperation mechanisms and actively introduce advanced technology and management experience to improve the capability of independent innovation continuously. Governments should incorporate knowledge related to GB into the continuing education curriculum for professionals and technicians, drive cooperation in GB-related technical fields and expand technical communication.

#### 3.3.4. Improve Evaluation and Strengthen Target Assessment

The country should establish a mechanism to assess and inspect the implementation of specific plans, refine and implement key work of GB, and dynamically monitor the completion of crucial indicators around the country.

#### 3.3.5. Innovate the Project Quality Supervision Mode

During the stages of planning, design, construction, and complete acceptance of new construction, the stakeholders should strengthen the supervision of the implementation of building energy efficiency and GB standards and encourage the use of “Internet + Supervision” to improve the efficiency of supervision.

#### 3.3.6. Play the Role of Market and Cultivate Industrial Support

The central government should guide building energy efficiency service institutions to provide market services for building operation and energy efficiency in existing residential buildings. To cultivate industrial support, governments should foster relevant industrial chains adapted to the development of high-quality GBs and promote technological upgrading and structural adjustment on the supply side of the industry.

### 3.4. Spatial Analysis of GB Development

The spatial analysis conducted in this section is divided into two parts. First, the common goals among those presented in Section 3.1 are extracted, and the different indicators of the common goals are presented in the form of bar charts to obtain a visual comparison. There is no goal that covers all 26 regions selected in this paper, so the common goals are those presented in most regions (over five). Secondly, individual goals with regional characteristics are analyzed separately to obtain the spatial characteristics of GB goals for the 14th Five-Year Plan and then find out what the regions are focusing on in the next five years in GB development.

#### 3.4.1. Spatial Analysis of Common Goals

When selecting the common development goals for most regions, this study screened out the proportional indicators from the national plan first and used them as a reference for the average level. Due to the limited number of regions selected in this study, it is impossible to compare the aggregate indicators in the national plan. Although some of the common objectives do not cover all of the regions selected in this study due to their different presentations, it does not mean that regions have no development requirement for these common objectives. To maintain consistency in the presentation of development objectives across the regions, the study has tried to express similar objectives in the same form as much as possible, based on thorough research and understanding of the documents.

##### Analysis of 100% Indicators

The above figures (Figure 2, Figure 3 and Figure 4) show that there are several 100% targets for the GB development among 26 regions, and they have been organized in Table 2. Most of these indicators are used to describe the proportion of GBs and the standards applied to GB. Moreover, “Proportion of new urban-rural GBs” or “Proportion of new buildings in urban-rural areas that implement GB standards” are frequently mentioned. As one of the key methods to help the construction industry achieve carbon peaks and carbon neutrality goals, GBs should be put into practice, and GB standards can better assist in the construction of GBs.

In addition to the above regions, other regions also have indicators called “Proportion of new urban-rural GBs”, but their indicator values are not 100%, such as 98% in Fujian Province, 80% in Hainan Province, and 60% in Inner Mongolia. The proportion of new urban–rural GBs in Fujian is nearly 100% which can be given no special consideration. However, it is clear from the geographical location that Hainan Province is surrounded by sea on all sides, and Inner Mongolia is deep inland. The special geographical location and extreme climatic conditions will certainly make the development of their construction industry relatively lagging, and the introduction of GB-related industries and technologies will also be difficult [51]. At the same time, due to the level of economic development, the incentive mechanism for GBs in Hainan and Inner Mongolia has not yet been improved, and the existing incentive mechanism is relatively single compared to some advanced regions, which cannot better guide the development of GB.

Some regions like Beijing also have expressed in other ways that the region will adopt 100% GBs for new construction and 100% GB standards, referring to the “full implementation of green standards” mentioned in “The implementation Plan for the GB Initiative in Beijing (2020–2022)” issued on 3 June 2021.

##### Application of Renewable Energy

**Figure 6:**
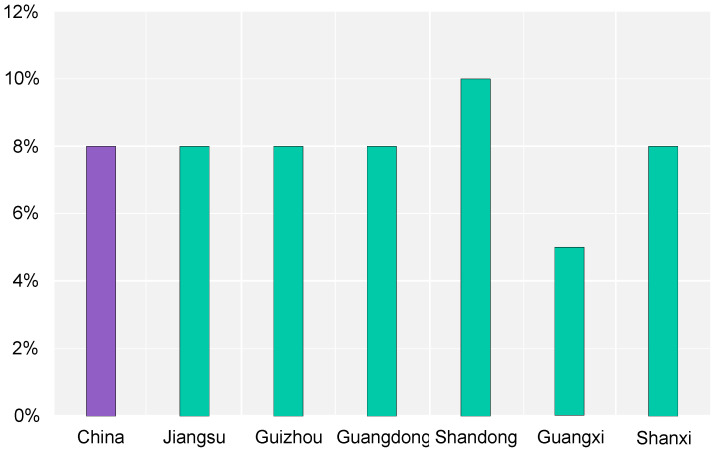
Replacement rate of renewable energy in urban–rural buildings.

The replacement rate of renewable energy in urban buildings is the ratio of the amount of renewable energy to the total consumption of primary energy. In Figure 6, six regions have specific targets for the replacement rate of renewable energy in urban buildings, which do not differ too much from each other, while Shandong’s target of 10% is above the national average level and Guangxi’s target of 5% is below the national level. Although other regions have not specifically indicated the expected targets for the replacement rate of renewable energy in urban buildings in their development targets, they do not ignore the development of renewable energy. Inner Mongolia set 30% for the application ratio of renewable energy in civilian buildings, while Chongqing will establish the technical path of implementation of renewable energy and try to achieve the construction of geothermal energy and air thermal energy building application areas of 5 million square meters at the end of the 14th Five-Year Plan.

**Figure 7:**
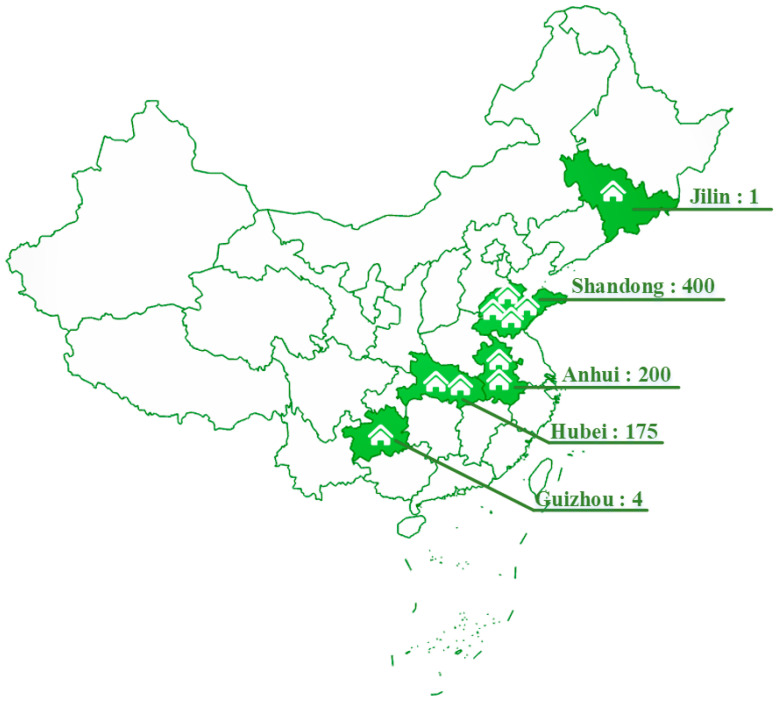
Area of application of renewable energy building (million square meters).

Except for the replacement rate of renewable energy in urban–rural buildings, some regions have also set targets for the area of application of renewable energy building (Figure 7). Several regions with larger indicator values, such as Shandong, Anhui, and Hubei, located in the east, may have higher requirements for renewable energy applications based on their more advanced level of economic development.

##### Energy Efficiency Retrofitting of Existing Buildings

**Figure 8:**
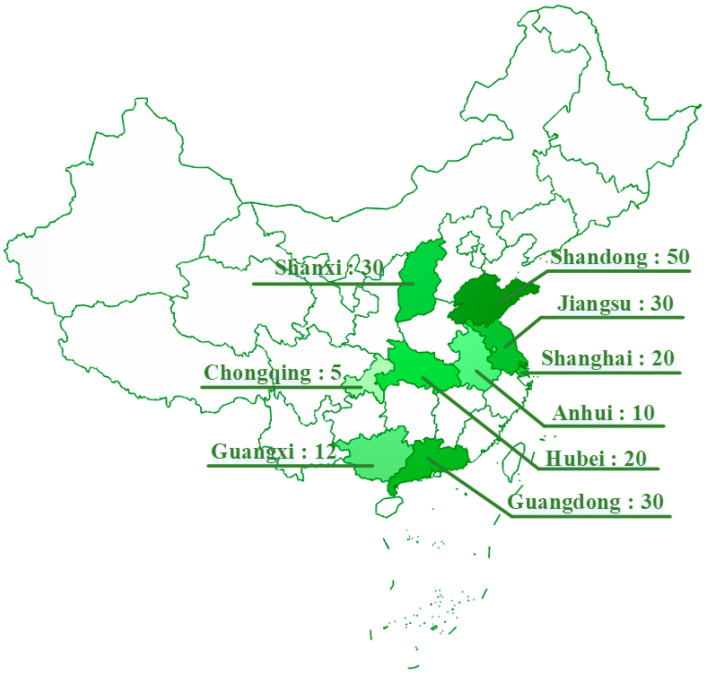
Area of energy efficiency retrofitting of existing buildings (million square meters).

Nowadays, many existing buildings cannot reach the energy standards because they were built a long time ago [52], and their carbon emissions are one of the main sources of carbon emissions in China’s construction sector. It is essential to promote energy efficiency by retrofitting existing buildings, which is one of the most important ways to achieve carbon reduction and resource conservation. To better improve the energy efficiency of existing buildings, the Chinese government has introduced several building energy retrofit policies from 2002–2017 to help implement energy efficiency retrofitting [53]. Nine regions selected in this paper have clear targets for energy efficiency retrofitting of existing buildings in the “14th Five-Year Plan” period (Figure 8). Regions with higher target values are mostly located in the eastern coastal region. The overall distribution of indicators shows that energy efficiency retrofitting is not promoted well nationwide. Although some regions have set high target values, the national average is still not very high.

Identifying the constraints that currently hinder the development of energy efficiency retrofitting in China is the first step for their implementation. The lack of capital investment and lack of novel technologies should be resolved through the exchange of economic development and technology [54]. The most basic issue for governments is the willingness of households to accept energy efficiency retrofitting. Previous studies found that significant differences existed in the views of energy efficiency retrofitting among residents with different characteristics [55]. Timely and appropriate measures to increase the level of willingness of residents are one of the prerequisites for achieving energy efficiency retrofitting.

##### Construction of Ultra-Low Energy Buildings

**Figure 9:**
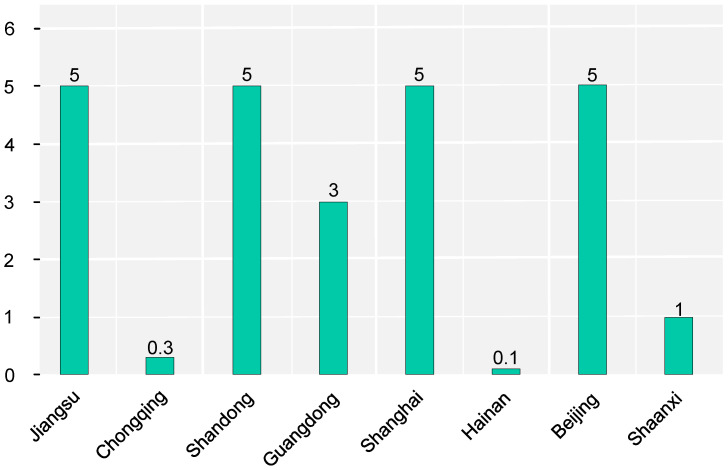
Area of construction of ultra-low energy buildings (million square meters).

Some scholars have found that special climatic conditions, such as high temperatures than in other regions [56] and special geological conditions referring to slopes over 5% and undulating terrain [57], all make it more difficult for Chongqing to build ultra-low energy buildings, and so do Hainan. This can explain why neither Chongqing nor Hainan has target values for the area of construction of ultra-low energy buildings (Figure 9).

##### Application of GB Materials

**Figure 10:**
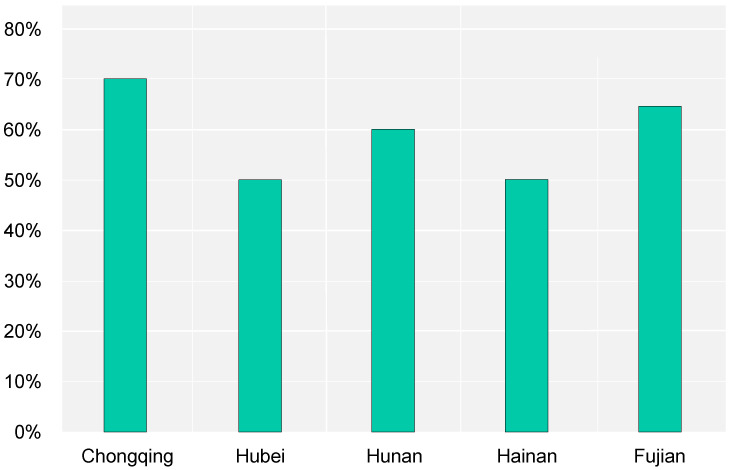
Proportion of application of GB materials in new buildings in the urban–rural area.

In 2014, the MOHURD officially defined that GB materials can reduce the consumption of natural resources and mitigate ecological impacts throughout their life cycle, which is issued in the “Management Measures of GB Materials Evaluation Label”. However, the use of poor-quality construction materials with a harmful environmental impact is a common occurrence in civil engineering construction [58]. Therefore, accelerating the use of GB materials can not only implement the concept of sustainable development but also improve the building process efficiency and construction quality. Due to the low utilization rate, high cost, and few supporting policies, the overall level of application of GB materials in China is lower than in some developed countries like Germany and the USA [59]. Thus, the target values for the proportion of GB materials shown in Figure 10 are relatively even and do not differ too much from each other. In “the 14th Five-Year Plan for the Development of Energy Efficiency and GB”, China has proposed to increase investment in the R&D of GB materials and key technologies and to take the lead in adopting GB materials in government-invested projects, aiming to increase the application rate of GB materials.

#### 3.4.2. Spatial Analysis of Targets with Regional Characteristics

In this section, this study analyzes the development targets with regional characteristics, such as the proportion of bulk cement used in Guangdong and the cumulative consumption of phosphogypsum in the construction industry in Guizhou. In particular, concerning the application of renewable energy, the application of solar energy varies greatly from region to region, mainly due to their geographical location, and will be discussed in detail.

##### Bulk Cement Use in Guangdong

At the end of the 20th century, the Guangdong Provincial People’s Government took into account its actual situation and formulated the Guangdong Province Bulk Cement Regulations, aiming to increase the proportion of bulk cement use, develop commercial concrete, save energy and raw materials, reduce environmental pollution, promote technological progress in cement production, and guarantee the quality of construction projects. To promote the further development of bulk cement, the Guangdong Provincial Department of Housing and Urban–Rural Development issued “the Guangdong Province Bulk Cement Development and Application Plan (2014–2020)” in 2014. The Plan stated that the province’s bulk cement supply should reach 95.3 million tons by the end of 2020, with a bulk rate of over 70%. Actually, the supply of bulk cement reached 103 million tons, and the cement bulk rate reached 72.51% in 2020, with an increase of 8.25% year-on-year, successfully completing the target in 2014. Among the 26 regions selected in this study, only Guangdong has a clear focus on the development of bulk cement in its planning document, and the provincial government has issued several decrees and regulations to ensure the stable development of bulk cement. This is a feature of the 14th Five-Year Plan for GB development in Guangdong.

##### Cumulative Consumption of Phosphogypsum (PG) in the Construction Industry in Guizhou

China generates almost 20 million tons of PG every year, and its utilization ratio is less than 10% [60]. When a large amount of PG is thrown away without any treatment, it can contaminate the environment [61]. The pollution of the Wujiang River in Guizhou is due to the incorrect handling of PG. To alleviate PG pollution in the Wujiang River, the Guizhou government has proposed a target for cumulative consumption of PG and has stated in its planning document that it will accelerate the R&D of building material products for the comprehensive use of PG resources and increase the promotion and application of PG building material products. Moreover, PG can be reasonably used in different fields, such as building materials, the chemical industry, and the recovery and extraction of rare earth elements [62].

##### Analysis of Solar Applications in Different Regions

China’s total solar radiation resources are abundant. The Qinghai–Tibet Plateau is the most abundant, with total annual radiation exceeding 1800 kWh/m^2^ and even over 2000 kWh/m^2^ in some areas. The Sichuan Basin has relatively low resources, with areas below 1000 kWh/m^2^. Table 3 shows the distribution of solar resources in the country. These data are from the National Energy Administration [63].

The distribution of solar resources influences the application of solar energy in various regions. Only a few of the regions selected in this study mentioned the application of solar energy resources (Table 4). Jiangsu, Guangdong, Guangxi, and Hubei all mentioned the newly installed capacity of solar photovoltaic in buildings, and Hubei’s indicator value is the lowest, as parts of Hubei are in the general zone of distribution of solar resources. Although Jiangsu, Guangdong, and Guangxi are all included in the “Richer zone”, Jiangsu has a lower target for the newly installed solar photovoltaic capacity in buildings compared to Guangdong and Guangxi. However, Jiangsu does not reduce the use of its abundant solar resources; it has separately proposed an indicator called the area of application of new solar thermal buildings. However, some regions such as Chongqing and Sichuan have no requirements for solar energy application in their planning documents, as the total annual radiation is much lower than in other regions, but they are committed to developing other renewable energy resources such as geothermal energy.

## 4. Discussion and Suggestion

The results show that there are spatial imbalances in the development goals of each region in the “14th Five-Year Plan” period. Development goals are proposed based on the current development situation, which can reflect the development situation to some extent. Therefore, the spatial imbalances in the development goals of GB in China suggest spatial imbalances in the development situation of GB in China. This finding aligns with the findings of Guo and Yuan [64] and Chen et al. [65]. In other words, regions with better GB development are more likely to have more optimistic development goals, while there is relatively little room to move forward in GB development for regions with a poorer economic situation.

The objective of this study is to identify the characteristics of GB development goals in each region during the “14th Five-Year Plan” period. After analyzing common goals and goals with regional characteristics, the results also reflect that the variability between many of the indicators may be arguably due to the level of economic and technical conditions or the special geographical location [66]. Concerning the proportion of new urban–rural GBs and the proportion of new buildings in urban–rural areas that implement GB standards, many regions have set a target value of 100%, reflecting that the majority of regions will promote GB development widely. However, some regions cannot achieve the target of 100% due to their economic level and geographical location, which requires them to exchange technology with regions better developed [67]. There is little variation between regions in the replacement rate of renewable energy in urban–rural buildings. The regions with a larger area of application of renewable energy building are mostly located in eastern China. Moreover, regions with a larger area of energy efficiency retrofitting of existing buildings are located in the eastern coastal region. Namely, the eastern coast has clear development goals and promising development prospects for renewable energy applications and energy efficiency retrofitting [68]. For the application of GB materials, the result is in line with findings in the research of Zhao et al. [59] that the overall level of application of GB materials in China is not very high compared to that of developed countries. When analyzing targets with regional characteristics, it indicates that Guangdong focuses on developing bulk cement in order to protect the environment; Guizhou proposes a rational application of PG to reduce pollution of the Wujiang River and the utilization of solar energy is related to the geographical location of regions.

The development barriers and paths concluded from the planning documents partially overlap with the existing research findings [9,50,69]. In the planning documents, there are several problems for GB development, such as poor quality of GB development, low level of prefabricated building development, insufficient awareness and lack of sense of acquisition for GB, an imperfect market mechanism for GB, and a weak foundation for the development of GB material industry. It is in line with previous research that immature market mechanisms for GB [70] and inappropriate development approaches [71] can also hinder GB development. Because the 14th Five-Year Plan has just been put into practice, the development barriers describing what the regions faced at the end of the “13th Five-Year Plan” can be applicable to the current stage. Simultaneously, to guarantee the stable development of GB, the national and regional governments play a pivotal role in promoting GB development [72]. Specifically, the primary task is to promote scientific and technological innovation and strengthen cooperation and communication between each region. Regions with advanced GB development can provide technical and strategic support to help achieve a breakthrough in technological bottlenecks, and technology transfer is critical. Technology transfer can be defined as an activity centered on knowledge exchange, and several technology transfer instruments have been proposed to apply in different areas. Among these, the triple helix of governments–universities–industries can promote innovation and may also be applicable to the green building industry in China [73]. Secondly, national and regional governments should formulate laws and regulations to guarantee the stable development of GB from the perspective of policy. Appropriate financial incentive policies should be proposed to motivate concerned organizations to take an active part in GB construction. Finally, it is essential to increase public acceptance of GB and consolidate the mass base for promoting GB development.

However, when analyzing common indicators, it is difficult to find a common indicator covering both the country and all the regions, and this is where some improvements can be made. When the country sets national development targets, it should simultaneously issue blank development documents containing basic and compulsory indicators to the regional governments. The regional governments or the Department of Housing and Urban–Rural Development should thoroughly consider the situation in the region and define the corresponding development targets, such as proportion or aggregate targets. After that, they should supplement the development targets with regional characteristics accordingly.

## 5. Conclusions

As global warming intensifies, all fields should find a sustainable direction for low-carbon development. GB, which consumes fewer resources and energy and produces less pollution, is the path to realizing sustainable development in the building sector. This study collected GB development planning documents that have been issued and put into practice in 26 regions since the implementation of the 14th Five-Year Plan. This paper conducted a qualitative analysis and concluded the development targets of these regions. Common targets and individual targets with regional characteristics have been analyzed. It can be found that there are spatial differences in the GB development targets in each region, and the development priorities also vary from region to region.

This study contributes to both theoretical and practical implementation, which provides strong evidence to verify the imbalanced spatial distribution of GB development goals in China and also provides regional governments with a relatively clear self-positioning to judge whether the current development level in each region keeps pace with the national development level. There are still some limits to this research, which can be a direction for further study. As not all regions have issued GB planning documents, the study does not cover all provinces and autonomous regions, and it may lead to deviations in the results to some extent. In addition, it should be not only an analysis of future targets but also a validation of rationality when setting goals combined with the regional situation. Future similar studies could be carried out on a cyclical basis (coinciding with the national Five-Year Plan) and study the state of national GB development periodically, then propose a focus and path of development for the next phase.

## Figures and Tables

**Table 1:** National GB development goals.

Key Indicators	Target 2025
Total consumption of secondary energy for building operating once (billion tons of standard coal)	11.5
Proportion of new residential buildings with improved energy efficiency levels in the urban–rural area	30%
Proportion of new public buildings with improved energy efficiency levels in urban–rural area	20%
Area of energy efficiency retrofitting of existing buildings (billion square meters)	0.35
Area of construction of ultra-low and nearly-zero energy buildings (billion square meters)	0.05
Proportion of prefabricated buildings to new urban buildings	30%
The new installed capacity of solar photovoltaic in buildings (billion kWh)	0.05
Area of application of new geothermal energy building (billion square meters)	0.1
Replacement rate of renewable energy in urban buildings	8%
Proportion of electricity consumption in building energy consumption	55%

**Table 2:** Key indicators with a target value of 100%.

Region	Key Indicators with Target Value of 100%
Jilin	Proportion of new urban–rural GBs
Tianjin	Proportion of new urban–rural GBs
Anhui	Proportion of new urban–rural GBs
Qinghai	Proportion of new urban–rural GBs
Jiangxi	Proportion of new urban–rural GBs
Shaanxi	Proportion of new urban–rural GBs
Hebei	Proportion of new urban–rural GBs
Sichuan	Proportion of new urban–rural GBs
Guizhou	Proportion of new urban–rural GBs
Chongqing	Proportion of new urban–rural GBs
Jiangsu	Proportion of new buildings in urban–rural areas that implement GB standards
Hubei	Proportion of new buildings in urban–rural areas that implement GB standards
Anhui	Proportion of new buildings in urban–rural areas that implement GB standards
Inner Mongolia	Proportion of new buildings in urban–rural areas that implement GB standards
Chongqing	Proportion of implementation of building energy efficiency measures for new buildings in the urban–rural area
Gansu	Implementation rate of mandatory design and construction standards for energy efficiency in new urban–rural buildings
Qinghai	Proportion of new urban–rural residential buildings that implement energy efficiency standards of 75% or higher

**Table 3:** Total national solar radiation and regional distribution.

Name	Total Annual Radiation MJ/m^2^	Major Regions
The richest zone	≥6300	West of Ejin Banner in Inner Mongolia, west of Jiuquan in Gansu, most of west of 100° E in Qinghai, most of west of 94° E in Tibet, eastern edge of Xinjiang, part of Ganzi in Sichuan
Very rich zone	5040~6300	Most of Xinjiang, most of east of Ejin Banner in Inner Mongolia, western Heilongjiang, western Jilin, western Liaoning, most of Hebei, Beijing, Tianjin, eastern Shandong, most of Shanxi, northern Shaanxi, Ningxia, most area of east of Jiuquan in Gansu, eastern edge of Qinghai, east of 94° E in Tibet, central and western Sichuan, most of Yunnan, Hainan
Richer zones	3780~5040	North of 50° N in Inner Mongolia, most of Heilongjiang, east-central Jilin, east-central Liaoning, west-central Shandong, southwestern Shanxi, south-central Shaanxi, eastern edge of Gansu, central Sichuan, eastern edge of Yunnan, southern Guizhou, most of Hunan, most of Hubei, Guangxi, Guangdong, Fujian, Jiangxi, Zhejiang, Anhui, Jiangsu, Henan
General zone	<3780	Eastern Sichuan, most of Chongqing, north central Guizhou, west of 110° E in Hubei, northwestern Hunan

**Table 4:** Development indicators related to solar energy.

Region	Key Indicator	Target 2025
Jiangsu	Newly installed capacity of applications of solar photovoltaic integration (kWh)	500
Jiangsu	Area of application of new solar thermal building (million square meters)	50
Guangdong	Newly installed capacity of solar photovoltaic in buildings (million kWh)	2
Guangxi	Newly installed capacity of solar photovoltaic in buildings (million kWh)	1.5
Hubei	Applications of solar photovoltaic building (kWh)	300

## Data Availability

The data used to support the findings of this study are available from the corresponding author upon request.

## References

[1] Li X., Damartzis T., Stadler Z., Moret S., Meier B., Friedl M., Marechal F. (2020). Decarbonization in Complex Energy Systems: A Study on the Feasibility of Carbon Neutrality for Switzerland in 2050. Front. Energy Res..

[2] Shen Y.Y., Faure M. (2021). Green building in China. Int. Environ. Agreem. Politics Law Econ..

[3] Central People’s Government of the People’s Republic of China (2022). Xi Jinping Delivers Important Speech at the General Debate of the 75th Session of the UN General Assembly. http://www.gov.cn/xinwen/2020-09/22/content_5546168.htm.

[4] Wu Z.Z., Jiang M.Y., Cai Y.Z., Wang H., Li S.H. (2019). What Hinders the Development of Green Building? An Investigation of China. Int. J. Environ. Res. Public Health.

[5] Xi C., Cao S.J. (2022). Challenges and Future Development Paths of Low Carbon Building Design: A Review. Buildings.

[6] Bungau C.C., Bungau T., Prada I.F., Prada M.F. (2022). Green Buildings as a Necessity for Sustainable Environment Development: Dilemmas and Challenges. Sustainability.

[7] Doan D.T., Ghaffarianhoseini A., Naismith N., Zhang T., Ghaffarianhoseini A., Tookey J. (2017). A critical comparison of green building rating systems. Build. Environ..

[8] Luo X., Liu H., Zhao X., Mao P. (2022). Managing the additional cost of passive buildings from the supply chain perspective: A case of Nanjing, China. Build. Environ..

[9] Li Z.D., IOP Research on risk Management of green Building development. Proceedings of the 6th International Conference on Advances in Energy Resources and Environment Engineering (ICAESEE).

[10] Zhang Y., Kang J., Jin H. (2018). A Review of Green Building Development in China from the Perspective of Energy Saving. Energies.

[11] Central People’s Government of the People’s Republic of China (2021). Outline of the 14th Five-Year Plan for National Economic and Social Development of the People’s Republic of China and Vision in 2035. http://www.gov.cn/xinwen/2021-03/13/content_5592681.htm.

[12] Fan K., Wu Z.Z. (2020). Incentive mechanism design for promoting high-level green buildings. Build. Environ..

[13] Hwang B.G., Tan J.S. (2012). Green building project management: Obstacles and solutions for sustainable development. Sustain. Dev..

[14] Khoshbakht M., Gou Z.H., Lu Y., Xie X.H., Zhang J. (2018). Are green buildings more satisfactory? A review of global evidence. Habitat Int..

[15] Zhang Y.Q., Wang H., Gao W.J., Wang F., Zhou N., Kammen D.M., Ying X.Y. (2019). A Survey of the Status and Challenges of Green Building Development in Various Countries. Sustainability.

[16] Song Y., Li C.S., Zhou L., Huang X.R., Chen Y., Zhang H.X. (2021). Factors affecting green building development at the municipal level: A cross-sectional study in China. Energy Build..

[17] Wu Z.Z., He Q.F., Yang K.J., Zhang J.M., Xu K.X. (2021). Investigating the Dynamics of China’s Green Building Policy Development from 1986 to 2019. Int. J. Environ. Res. Public Health.

[18] Zou Y.H., Zhao W.X., Zhong R.J. (2017). The spatial distribution of green buildings in China: Regional imbalance, economic fundamentals, and policy incentives. Appl. Geogr..

[19] Kaza N., Lester T.W., Rodriguez D.A. (2013). The Spatio-temporal Clustering of Green Buildings in the United States. Urban Stud..

[20] Gao Y., Yang G.S., Xie Q.H. (2020). Spatial-Temporal Evolution and Driving Factors of Green Building Development in China. Sustainability.

[21] Zou P.X.W., Sunindijo R.Y., Dainty A.R.J. (2014). A mixed methods research design for bridging the gap between research and practice in construction safety. Saf. Sci..

[22] Zou P.X.W., Xu X.X., Sanjayan J., Wang J.Y. (2018). A mixed methods design for building occupants’ energy behavior research. Energy Build..

[23] Ministry of Housing and Urban-Rural Development of the People’s Republic of China (2022). The 14th Five-Year Plan for the Development of Energy Efficiency and Green Building. https://www.mohurd.gov.cn/gongkai/fdzdgknr/zfhcxjsbwj/202203/20220311_765109.html.

[24] Department of Housing and Urban-Rural Development of Guizhou Province (2022). The 14th Five-Year Plan for the Development of Construction Technology and Green Building in Guizhou Province.

[25] Department of Housing and Urban-Rural Development of Anhui Province (2022). The 14th Five-Year Plan for the Development of Building Energy Conservation and Green Building in Anhui Province. http://dohurd.ah.gov.cn/public/6991/56587531.html.

[26] Department of Housing and Urban-Rural Development of Shandong Province (2022). The 14th Five-Year Plan for the Development of Green Building and Building Energy Efficiency in Shandong Province. http://zjt.shandong.gov.cn/art/2022/4/13/art_102884_10306483.html.

[27] Department of Housing and Urban-Rural Development of Shanxi Province (2022). The 14th Five-Year Plan for Building Energy Conservation, Green Building and Science and Technology Standards in Shanxi Province. https://zjt.shanxi.gov.cn/zwgk/tfwj/202206/t20220630_6525305.shtml.

[28] Qinghai Provincial People’s Government (2021). The 14th Five-Year Plan for Urban and Rural Housing Development in Qinghai Province. http://www.qinghai.gov.cn/xxgk/xxgk/fd/ghxx/202201/t20220112_188880.html.

[29] Department of Housing and Urban-Rural Development of Hubei Province (2021). Implementation Opinions on Building Energy Conservation and Green Building Development in the “14th Five-Year Plan” of Hubei Province. http://zjt.hubei.gov.cn/zfxxgk/fdzdgknr/ghxx/202111/t20211130_3890946.shtml.

[30] Department of Housing and Urban-Rural Development of Guangdong Province (2022). The 14th Five-Year Plan for the Development of Building Energy Conservation and Green Building in Guangdong Province. http://zfcxjst.gd.gov.cn/gkmlpt/content/3/3905/post_3905996.html#1422.

[31] Department of Housing and Urban-Rural Development of Gansu Province (2022). The 14th Five-Year Plan for the development of Building Energy Conservation and Green Building in Gansu Province. http://zjt.gansu.gov.cn/zjt/c108287/202208/2114063.shtml.

[32] Tianjin Housing and Urban-Rural Development Commission (2021). The 14th Five-Year Plan for Green Building Development in Tianjin. https://zfcxjs.tj.gov.cn/xxgk_70/zcwj/wfwj/202105/t20210510_5446439.html.

[33] Department of Housing and Urban-Rural Development of Guangxi Zhuang Autonomous Region (2022). The 14th Five-Year Plan for the Development Building Energy Conservation and Green Building in Guangxi. http://zjt.gxzf.gov.cn/zfxxgk/fdzdgknr/wjtz/t12998044.shtml.

[34] Shanghai Housing and Urban-Rural Construction Management Committee (2021). The 14th Five-Year Plan for Green Building Development in Shanghai. https://zjw.sh.gov.cn/jsgl/20211109/d4b85b4ab0f94c108b58b01c27b1fa91.html.

[35] Department of Housing and Urban-Rural Development of Inner Mongolia Autonomous Region (2022). The 14th Five-Year Plan for the Development of Building Energy Conservation and Green Building in Inner Mongolia Autonomous Region. http://zjt.nmg.gov.cn/zwgk/zfxxgkn/fdzdgknr/bmwj/202208/t20220825_2117046.html.

[36] Department of Housing and Urban-Rural Development of Hunan Province (2022). The 14th Five-Year Plan for the Development of Building Energy Conservation and Green Building in Hunan Province. https://zjt.hunan.gov.cn/zjt/xxgk/xinxigongkaimulu/tzgg/tzgg2jzjnykj/202211/t20221104_29115764.html.

[37] Department of Housing and Urban-Rural Development of Hainan Province (2021). The 14th Five-Year Plan for Green Building (Prefabricated Building) Development in Hainan Province (2021–2025). http://zjt.hainan.gov.cn/szjt/zptzgg/202107/d429a4c44adf4093ba311ce6a4a0f792.shtml.

[38] Department of Housing and Urban-Rural Development of Jilin Province (2021). The 14th Five-Year Plan for the Development of Building Energy Conservation and Green Building in Jilin Province. http://xxgk.jl.gov.cn/zcbm/fgw_98022/xxgkmlqy/202207/t20220720_8516494.html.

[39] Department of Housing and Urban-Rural Development of Shaanxi Province (2021). The 14th Five-Year Plan for the Development of Housing and Urban-Rural Construction in Shaanxi Province. https://js.shaanxi.gov.cn/zcfagui/2021/9/114204.shtml?t=2020.

[40] Department of Housing and Urban-Rural Development of Sichuan Province (2021). The 14th Five-Year Plan Sichuan Province Construction Industry Development Plan. https://www.sc.gov.cn/10462/10778/10876/2021/9/26/db6df077008d4e76a790f3ca12f134af.shtml.

[41] Department of Housing and Urban-Rural Development of Yunnan Province (2021). The 14th Five-Year Plan for the Development of Green Prefabricated Building Industry in Yunnan Province. https://zfcxjst.yn.gov.cn/zhengfuwenjian8655/284647.html.

[42] Beijing Municipal Commission of Housing and Urban-Rural Development (2022). The 14th Five-Year Plan for the Development of Construction Industry in Beijing. http://zjw.beijing.gov.cn/bjjs/xxgk/ghjh/325907958/index.shtml.

[43] Department of Housing and Urban-Rural Development of Hebei Province (2021). The 14th Five-Year Plan for the Industrialization of New Buildings in Hebei Province. http://zfcxjst.hebei.gov.cn/hbzjt/zfxxgk/xxgknr/ghjh/101663762493534.html.

[44] Department of Housing and Urban-Rural Development of Fujian Province (2021). The 14th Five-Year Plan for the Development Construction Industry in Fujian. http://zjt.fujian.gov.cn/xxgk/zfxxgkzl/xxgkml/dfxfgzfgzhgfxwj/jzsc/202108/t20210818_5671949.htm.

[45] Department of Housing and Urban-Rural Development of Zhejiang Province (2021). The 14th Five-Year Plan for Housing and Urban-Rural Construction in Zhejiang Province. https://fzggw.zj.gov.cn/art/2021/5/8/art_1229123366_2283979.html.

[46] Department of Housing and Urban-Rural Development of Jiangxi Province (2021). The 14th Five-Year Plan for the Development of Housing Urban-Rural Construction in Jiangxi Province. http://zjt.jiangxi.gov.cn/art/2021/12/29/art_40687_3808581.html.

[47] Department of Housing and Urban-Rural Development of Heilongjiang Province (2021). The 14th Five-Year Plan for Construction Industry Development in Heilongjiang Province. http://zfcxjst.hlj.gov.cn/zfcxjst/c114765/202109/c00_31211397.shtml.

[48] Wang J.B., Jia Y. Analysis of the Development of China’s Green Building Space Difference Measure Based on the Theil Index. Proceedings of the International Conference on Social Science, Education Management and Sports Education (SSEMSE).

[49] Prum D.A., Aalberts R.J., Percio S.D. (2012). In Third Parties We Trust? The Growing Antitrust Impact of Third-Party Green Building Certification Systems for State and Local Governments. J. Envtl. L. Litig..

[50] Li Y.A., Yang L., He B.J., Zhao D.D. (2014). Green building in China: Needs great promotion. Sustain. Cities Soc..

[51] Gao C. (2018). Green Building Promotion Strategy Based on SWOT of Hainan Province. Build. Energy Effic..

[52] Li Y.T., Jun R., Zhou J., Lu J.P., Qing Y., Lv Z.J. The Existing Building Sustainable Retrofit in China-A Review and Case Study. Proceedings of the 10th International Symposium on Heating, Ventilation and Air Conditioning (ISHVAC).

[53] Liu G., Li X.H., Tan Y.T., Zhang G.M. (2020). Building green retrofit in China: Policies, barriers and recommendations. Energy Policy.

[54] Bao L.L., Zhao J., Zhu N. (2012). Analysis and proposal of implementation effects of heat metering and energy efficiency retrofit of existing residential buildings in northern heating areas of China in “the 11th Five-Year Plan” period. Energy Policy.

[55] Lai Y.P., Li Y.T., Feng X.Y., Ma T. (2022). Green retrofit of existing residential buildings in China: An investigation on residents’ perceptions. Energy Environ..

[56] Ding Y., Li B., Luo Q., Liu H., Liu M.J.F.o.A., China C.E.i. (2009). Effect of natural resource on improving indoor thermal environment in Chongqing. Front. Archit. Civ. Eng. China.

[57] Zhang X., Liu Y., Yang C. (2015). Quantifying spatial patterns of urban expansion in mountainous cities: The case of Chongqing. J. Southwest Univ. (Nat. Sci. Ed.).

[58] Sun P., Zhang X.S. Application of Green Building Materials in Civil Engineering Construction. Proceedings of the 3rd International Conference on Advances in Materials, Machinery, Electronics (AMME).

[59] Zhao H., Wang Y., Qiu W.T., Qu W.L., Zhang X.D., IOP Research on the Application of Green Building Materials in China. Proceedings of the International Conference of Green Buildings and Environmental Management (GBEM).

[60] Shen W.G., Zhou M.K., Zhao Q.L. (2007). Study on lime-fly ash-phosphogypsum binder. Constr. Build. Mater..

[61] Zhang H., Cheng Y., Yang L., Song W.K. (2020). Modification of Lime-Fly Ash-Crushed Stone with Phosphogypsum for Road Base. Adv. Civ. Eng..

[62] Wei Z.Q., Deng Z.B. (2022). Research hotspots and trends of comprehensive utilization of phosphogypsum: Bibliometric analysis. J. Environ. Radioact..

[63] National Energy Administration (2014). How Is Country’s Solar Energy Resources Distributed?. http://www.nea.gov.cn/2014-08/03/c_133617073.htm.

[64] Guo K., Yuan Y.B. (2021). Geographic Distribution and Influencing Factor Analysis of Green Residential Buildings in China. Sustainability.

[65] Chen L.Y., Gao X., Gong S.T., Li Z. (2020). Regionalization of Green Building Development in China: A Comprehensive Evaluation Model Based on the Catastrophe Progression Method. Sustainability.

[66] Hao T., Wang J. (2019). Study on Spatial Evolution of Chinese Green Building. IOP Conf. Ser. Earth Environ. Sci..

[67] Li J., Wu C.Y., Qu W.Y., Wang F., Li W.Z. (2013). Study on the Implementation and Development of the Green Building in China. Appl. Mech. Mater..

[68] Liu W., He Z., Chen H., Lin C. (2022). Comparative Analysis Chinese Green Buildings’ of Input–Output Effect Based on Data Envelope Analysis. Buildings.

[69] Zuo J., Zhao Z.Y. (2014). Green building research-current status and future agenda: A review. Renew. Sustain. Energ. Rev..

[70] Liu X.J., Hu W. (2019). Attention and sentiment of Chinese public toward green buildings based on Sina Weibo. Sustain. Cities Soc..

[71] Huo X.S., Yu A.T.W., Darko A., Wu Z.Z. (2019). Critical factors in site planning and design of green buildings: A case of China. J. Clean. Prod..

[72] Liu J.Y., Low S.P., He X. (2012). Green practices in the Chinese building industry: Drivers and impediments. J. Technol. Manag. China.

[73] Craiut L., Bungau C., Bungau T., Grava C., Otrisal P., Radu A.F. (2022). Technology Transfer, Sustainability, and Development, Worldwide and in Romania. Sustainability.

